# Transcriptional profile changes after treatment of ischemia reperfusion injury-induced kidney fibrosis with 18β-glycyrrhetinic acid

**DOI:** 10.1080/0886022X.2022.2061998

**Published:** 2022-06-14

**Authors:** Yamei Jiang, Chengzhe Cai, Pingbao Zhang, Yongsheng Luo, Jingjing Guo, Jiawei Li, Ruiming Rong, Yi Zhang, Tongyu Zhu

**Affiliations:** a Department of Urology, Zhongshan Hospital, Fudan University, Shanghai, P. R. China; b Shanghai Key Laboratory of Organ Transplantation, Shanghai, P. R. China; c Department of General Surgery, Zhongshan Hospital, Fudan University, Shanghai, P. R. China; d Biomedical Research Center, Institute for Clinical Sciences, Zhongshan Hospital, Fudan University, Shanghai, P. R. China

**Keywords:** 18β-Glycyrrhetinic acid, kidney, fibrosis, ischemia reperfusion injury, transcriptional profile, RNA-seq

## Abstract

**Introduction:**

Chronic kidney disease (CKD) is characterized by renal fibrosis without effective therapy. 18β-Glycyrrhetinic acid (GA) is reported to have detoxification and anti-inflammatory functions and promotes tissue repair. However, the role of GA in CKD remains unclear. In this study, we investigated whether GA has a potential therapeutic effect in kidney fibrosis.

**Methods:**

A renal fibrosis mouse model was established by ischemia/reperfusion (I/R) injury via clamping unilateral left renal pedicle for 45 min; then, the mice were treated with vehicle or GA. Kidney tissues and blood samples were extracted 14 days after reperfusion and renal function, histopathological staining, quantitative PCR, and western blotting were performed. RNA-seq was performed to explore the changes in the transcriptional profile after GA treatment.

**Results:**

Renal function, pathological and molecular analysis displayed that fibrosis was successfully induced in the I/R model. In the GA treatment group, the severity of fibrosis gradually reduced with the best effect seen at a concentration of 25 mg kg ^−1^. A total of 970 differentially expressed genes were identified. Pathway enrichment showed that reduced activation and migration of inflammatory cells and decreased chemokine interaction in significant pathways. Protein–protein interaction networks were constructed and 15 hub genes were selected by degree rank, including chemokines, such as C3, Ccl6, Ccr2, Ptafr, Timp1, and Pf4.

**Conclusions:**

GA may alleviate renal fibrosis by inhibiting the inflammatory response. GA is a promising therapy that may perhaps be used in treating renal fibrosis and CKD.

## Graphical Abstract

**Figure:**
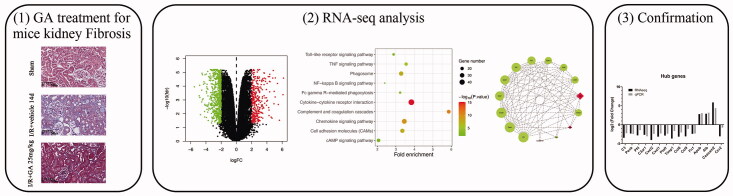

## Introduction

According to a 2017 global epidemiological survey, the worldwide prevalence of chronic kidney disease (CKD) is about 9.1%. Death caused by CKD or impaired renal function accounted for 4.6% of all deaths in 2017, and CKD was the twelfth leading cause of death [1]. The immense public health burden of CKD has aroused great interest in research into the mechanism and potential therapy of this disease worldwide. In clinical settings, kidney ischemia and reperfusion (I/R) injury leaded acute kidney injury is a common complication of major surgery such as cardiac surgery (accounting for 20%) and abdominal surgery [2,3]. Furthermore, it is also a common reason for delayed graft function after kidney transplantation [4]. I/R injury eventually results in serious adverse outcome of progressing to CKD [5]. Renal fibrosis with deposition of collagens and other extracellular matrix proteins is the basic feature of CKD. Targeting cytokines, transcription factors, developmental, and signaling pathways has been shown to ameliorate kidney fibrosis in preclinical studies [6]. In addition, experts in various fields have collaborated to provide insights into novel strategies to prevent and treat CKD through animals and biomimetic studies [7]. In the current post-genomic era, the application of systems biology enables in-depth analysis of CKD at the molecular and subcellular level [8]. It is imperative to explore promising effective therapeutic strategies for CKD.

Licorice is the most widely used herbal medicine in the world, and is grown in China, Japan, and Russia [9]. 18β-Glycyrrhetinic acid (GA) is the most well-known active metabolite of licorice, which has multiple functions; it not only has detoxification properties [10], but also possesses antioxidant and anti-inflammatory [11,12], immunoregulatory [13,14], and liver-protective [15–17] functions. Previous studies have reported that GA plays a role in kidney disease. The study by Abd El-Twab et al. [18] showed that GA reduced methotrexate-induced nephrotoxicity by upregulating the mRNA of nuclear factor-erythroid 2-related factor 2 (Nrf2) and hemoxygenase 1 (HO-1) in the kidney, which is related to attenuating oxidative stress and inflammation. Ma et al. [19] established a cisplatin-induced acute kidney injury (AKI) mouse model and demonstrated that GA inhibited renal tubular epithelial cell apoptosis by targeting histone deacetylases 2 (HDAC2) and enhancing the level of bone morphogenetic protein-7 (BMP-7) epigenetically. Furthermore, GA is also a very promising anti-fibrotic treatment for patients with chronic liver disease. Wang et al. demonstrated that GA downregulated the mRNA expression of type I and III pre-collagen and reduced the deposition of collagen in fibrotic rat liver [20]. However, no study has investigated the role of GA in ameliorating kidney fibrosis *in vivo*. Hence, the present study aimed to uncover the potential protective effect of GA in kidney fibrosis and explore its underlying mechanisms.

In the present study, we employed RNA-seq combined with bioinformatics to explore the differential gene expression in mice with kidney fibrosis with or without GA treatment in order to identify potential signaling pathways and candidate genes, which may be correlated with GA efficacy in kidney fibrosis.

## Materials and methods

### Animal models and treatment

All animal experiments were conducted according to the Care and Use of Laboratory Animals of Fudan University. The study protocol was approved by the Animal Ethical Committee of Zhongshan Hospital, Fudan University. Male C57BL/6 mice, 6- to 8-week old (20–23 g), were purchased from JSJ Laboratory Animals, Co., LTD (Shanghai, China) and were housed in a SPF-grade animal room. After one week accommodation, the mice were randomly divided into seven groups (*n* = 25): the vehicle sham group, the ischemia/reperfusion (I/R)+vehicle group, and the I/R + GA (5, 10, 25, 50, 75 mg kg^−1^) group.

For establishing the I/R-induced fibrosis model, C57BL/6 mice were anesthetized with pentobarbital intraperitoneally (i.p.). The depth of anesthesia was assessed through corneal and withdrawal reflexes. Then, unilateral left renal pedicle was clamped with a microaneurysm clamp (R31005-04, RWD Life Science Co., LTD, Guangzhou, China) for 45 min; 0.5 mL of 0.9% sterile saline was added to the abdomen during the ischemia time and the core temperature was kept between 36.5 °C and 37.5 °C during the whole procedure with an electronic heating pad. After removing the clamp, hemoperfusion was visible. For the sham model, the mice were subjected to all the procedures except clamping of the renal pedicle.

GA (G10105, Sigma-Aldrich, St. Louis, MO, USA) was dissolved in dimethyl sulfoxide (DMSO, D8371, Solarbio, Beijing, China) and diluted in corn oil to reach the usage concentrations of 5, 10, 25, 50, 75 mg kg^−1^. In each final usage concentration, DMSO accounted for 5% (w/v) in the corn oil. Mice were i.p. injected with 100 μL GA or vehicle (corn oil including 5% DMSO) 12 h before ischemia and received the same dose daily for 13 consecutive days after reperfusion. All mice were euthanized on day 14 after reperfusion. The blood, insulted left kidneys and the contralateral right kidneys were collected for further investigation.

### Renal function and liver function

Creatinine was tested (ml037726, Mlbio, Shanghai, China) using tissues of both insulted left kidney and the contralateral right kidney. In addition, mice serum was used to detected the systemic serum creatinine (SCr) (DICT-500, BioAssay System, Hayward, CA, USA), urea (DIUR-500, BioAssay System, Hayward, CA, USA), alanine aminotransferease (ALT) (ml063179, Mlbio, Shanghai, China), and aspartate aminotransferase (AST) (ml058577, Mlbio, Shanghai, China).

### Renal histopathology

One half of each insulted left kidney sample was fixed in 4% paraformaldehyde and embedded in paraffin, followed by sectioning at 4 μm thickness. Hematoxylin-Eosin (H&E), Masson, and Sirius red staining were performed to assess the degree and extent of fibrosis. Briefly, H&E staining was evaluated using tubular injury scores by two independent pathologists while the positive areas of Masson and Sirius red staining were quantified using ImageJ software (version 1.52a; National Institutes of Health, Bethesda, MD, USA), as described previously [21].

Immunohistochemistry (IHC) was conducted with primary antibodies against alpha-smooth muscle actin (α-SMA) (#19245, Cell Signaling Technology, Danvers, MA, USA) and fibronectin (ab268020, Abcam, Cambridge, UK). Positive area in IHC was calculated at ×400 magnification with ImageJ software.

IHC and immunofluorescence (IF) staining in protein level were also conducted for hub genes verification. IHC was performed with the indicated primary antibodies against C3 (ab200999, Abcam, Cambridge, UK), CCL6 (PA5-119021, ThermoFisher Scientific, Waltham, MA, USA), CCR2 (ab273050, Abcam, Cambridge, UK), PAFr (PA5-112676, ThermoFisher Scientific, Waltham, MA, USA), and TIMP1 (MA1-773, ThermoFisher Scientific, Waltham, MA, USA). IF staining was also conducted to verify PF4 (ab282111, Abcam, Cambridge, UK).

### RNA extraction and quantitative PCR

Total RNA was extracted using TRIzol (Invitrogen, Carlsbad, CA, USA) according to the manufacturer’s instructions. Reverse Transcription Kit (11141ES10, Yeasen, Shanghai, China) was used to obtain complementary DNA. Then, quantitative PCR (qPCR) was performed using SYBR Green Master Mix (11202ES03, Yeasen, Shanghai, China). The mRNA levels of target genes were normalized to the value of β-ACTIN mRNA in each sample and fold-change in expression was calculated using the 2^ΔΔCt^ method. The oligonucleotide primers (Sangon Biotech, Shanghai, China) for target genes are listed in Table 1.

**Table 1.** Primer sequence for qPCR analysis.

Genes (Mouse)	Forward	Reverse
*Acta2*	GTCCCAGACATCAGGGAGTAA	TCGGATACTTCAGCGTCAGGA
*Col1a1*	GAGGGCGAGTGCTGTGCTTTC	GGAGACCACGAGGACCAGAAGG
*C3*	CCAGCTCCCCATTAGCTCTG	GCACTTGCCTCTTTAGGAAGTC
*Penk*	TGCGCTAAATGCACGTACC	TCCCAGATTTTGAAAGAAGGCAG
*Pf4*	AGAGCCCTAGACCCATTTCCT	TGACATTTAGGCAGCTGATACC
*C3ar1*	TCGATGCTGACACCAATTCAA	TCCCAATAGACAAGTGAGACCAA
*Cxcl2*	CCAACCACCAGGCTACAGG	GCGTCACACTCAAGCTCTG
*Cxcl1*	CTGGGATTCACCTCAAGAACATC	CAGGGTCAAGGCAAGCCTC
*Ptafr*	TATACTGGGGGTGGTTGCCAA	GCAGGTCAGCCATAGTGAGATTC
*Timp1*	GCAACTCGGACCTGGTCATAA	CGGCCCGTGATGAGAAACT
*Ccl6*	GCTGGCCTCATACAAGAAATGG	GCTTAGGCACCTCTGAACTCTC
*Ccl9*	CCCTCTCCTTCCTCATTCTTACA	AGTCTTGAAAGCCCATGTGAAA
*Fn1*	GACCAGTGCCAAGATTCAGAGACC	TTCCTTCCAGCGACCCGTAGAG
*Apob*	AAGCACCTCCGAAAGTACGTG	CTCCAGCTCTACCTTACAGTTGA
*Alb*	TGCTTTTTCCAGGGGTGTGTT	TTACTTCCTGCACTAATTTGGCA
*Ceacam2*	TGGCAGAGAGGCACTATACAG	TGGAGTTGTTGCTTGTGATGT
*Ccr2*	ATCCACGGCATACTATCAACATC	CAAGGCTCACCATCATCGTAG

### Western blotting analysis

The insulted left kidney tissues were lysed with RIPA buffer (P0013C, Beyotime Biotechnology, Shanghai, China) supplemented with 1% phenylmethylsulfonyl fluoride (P0100, Solarbio, China). BCA protein assay kit (P0010, Beyotime Biotechnology, Shanghai, China) was used to detect protein concentration. The protein samples were separated in 10% sodium dodecyl sulfate polyacrylamide gel electrophoresis (SDS-PAGE) and then transferred to polyvinylidene difluoride (PVDF) membranes (IPFL00010, Millipore, Bedford, MA, USA). After being blocked in 5% nonfat milk at room temperature for 1.5 h, membranes were incubated with primary antibodies: anti-fibronectin (ab268020, Abcam, Cambridge, UK), anti-αSMA (#19245, Cell Signaling Technology, Danvers, MA, USA), and anti-COL1A1 (#91144, Cell Signaling Technology, Danvers, MA, USA) at 4 °C overnight. The expression levels of target proteins were normalized to that of β-actin (#8457, Cell Signaling Technology, Danvers, MA, USA). Afterward, the membranes were washed and incubated with horseradish peroxidase (HRP)-conjugated anti-rabbit/mouse IgG (L3012, SAB, Nanjing, China) at room temperature for 1.5 h. Enhanced chemiluminescence (ECL, 36208ES60, Yeasen, Shanghai, China) was used to visualize the blots.

### RNA-seq

For RNA-seq of tissue samples, insulted left kidney tissues from the vehicle sham group, the I/R + vehicle group, and the I/R + GA (25 mg kg^−1^) group (*n* = 3) were dissected and immediately placed in TRIzol reagent. Libraries were constructed and sequenced on the BGISEQ-500 platform (BGI, Shenzhen, China). Raw sequencing reads were cleaned by removing adaptor sequences, low-quality reads, and reads containing poly-N sequences using SOAPnuke (v1.5.2). Then, clean reads were mapped to the HISAT2 reference genome.

### Bioinformatics analysis

Normalization of raw data and differentially expressed genes (DEGs) analysis was performed using the ‘limma’ package in R software. |log FC| (fold change) >2 and *Q* value <0.05 were considered statistically significant. Heatmaps were visualized by using the ‘pheatmap’ package. All DEGs were put into the STRING database (http://string-db.org/) to predict the protein–protein interactions (PPI), with the species of *Mus musculus* and the highest confidence score was set at 0.9. To delve into the function and pathways of DEGs, the Gene ontology (GO) and the Kyoto Encyclopedia of Genes and Genomes (KEGG) enrichment analyses were performed using the ‘clusterProfiler’ package. GO analysis consisted of cellular component (CC), molecular function (MF), and biological processes (BP) analyses. Cytoscape software (version 3.8.0) was applied to construct PPI for hub genes, with confidence scores set at 0.4. Finally, the top 15 genes of the highest degree rank were selected for further verification.

### Statistical analysis

All data are presented as means ± *SEM*. Data analysis was conducted by using GraphPad Prism software (version 9.0.1, San Diego, CA, USA). One-way analysis of variance (ANOVA) was used to assess statistical significance between groups when data was in normal distribution, otherwise, nonparametric test was used. A *p*-value less than .05 was considered statistically significant.

## Results

### GA improved kidney function and alleviated renal fibrosis

**Figure 1.**
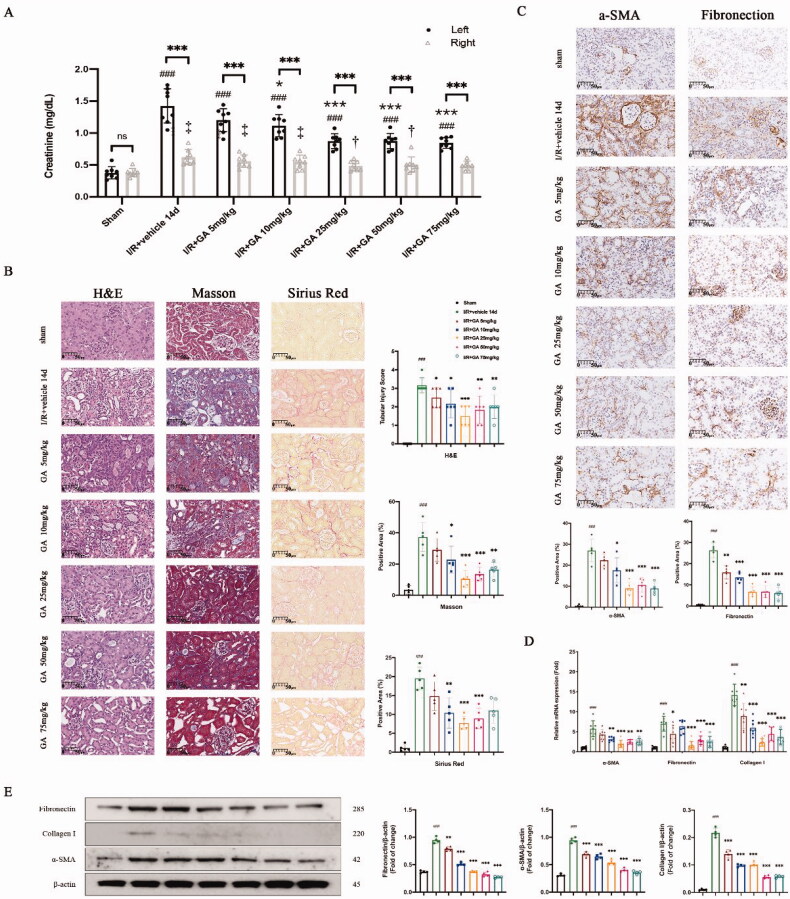
18β-GA attenuated I/R-induced mice renal injury and fibrosis. On day 14 after I/R injury, both the insulted left kidney and the contralateral right kidney were harvested for analysis. (A) Kidney function of both kidneys. ^###^*p* < .001 compared with left kidneys in Sham group; ^†^*p* < .05, ^‡^*p* < .01 compared with right kidneys in Sham group; *p < .05, ***p < .001 compared with left kidneys in I/R + vehicle 14d group; ^∗∗∗^*p* < .001 compared the left and right kidneys. (B) Representative images of H&E, Masson and Sirius Red staining (×400). (C) Representative images of IHC staining: α-SMA, Fibronectin (×400). Quantification of positive area by ImageJ. (D) mRNA expression confirmed the reduced fibrosis after GA treatment. (E) Western blot of Fibronectin, Collagen I and α-SMA expression. ^###^*p* < .001 compared with the Sham group. ^∗^*p* < .05, ^∗∗^*p* < .01, ^∗∗∗^*p* < .001 compared with the I/R + vehicle 14d group for (B–E).

Renal function test showed severe kidney injury in the insulted left kidneys but GA alleviated kidney injury. As shown in Figure 1(A), creatinine of the left kidney was much higher in I/R + vehicle 14d group compared with the sham group. After GA treatment, creatinine decreased significantly when GA dose was more than 10 mg/kg. For the contralateral right kidney, although the creatinine rose statistical significantly compared with the sham group, the level was much lower than those in the left kidney (*p* < .001). Systemic renal function displayed a totally different result. SCr and urea in I/R + vehicle 14 group increased after I/R injury but the ascending degree was lower than creatinine derived from the left kidney tissue. In addition, SCr and urea showed no decrease after GA treatment (Supporting Information Figure S1(A)). By the way, during the 14 days, all mice were alive and the ALT and AST had no fluctuation in all group (Supporting Information Figure S1(B)).

Two weeks after reperfusion, in comparison with the sham group, tubular injury and fibrosis in the I/R + vehicle group was obvious (*p* < .001) when stained with H&E, with dilated tubules, tubular disorganization, and extracellular matrix deposition. Administration of GA alleviated kidney fibrosis 14 days post-I/R injury. Kidney pathology displayed reduction of injury and fibrosis macroscopically; the extent of the decrease in fibrosis corresponded to increasing GA concentrations (Figure 1(B)). Compared to the I/R + vehicle group, tubular injury score and positive areas of Masson and Sirius red (that delineate fibrosis) were significantly lower in the I/R + GA group, especially in I/R + GA 25 mg kg^−1^ group. In addition, IHC staining revealed that the expression of α-SMA and fibronectin in the interstitial area were markedly lower when mice received GA up to 25 mg kg^−1^ or more compared to the I/R + vehicle group on the fourteenth day (Figure 1(C)). To further corroborate this, qPCR (Figure 1(D)) and western blotting (Figure 1(E)) were performed, and we found that the trend of these fibrosis markers corresponded with the histopathological results. These results indicate that kidney fibrosis was successfully established 14 days after I/R injury in mice, and GA had a therapeutic effect on kidney fibrosis.

### Identification of differentially expressed genes

**Figure 2.**
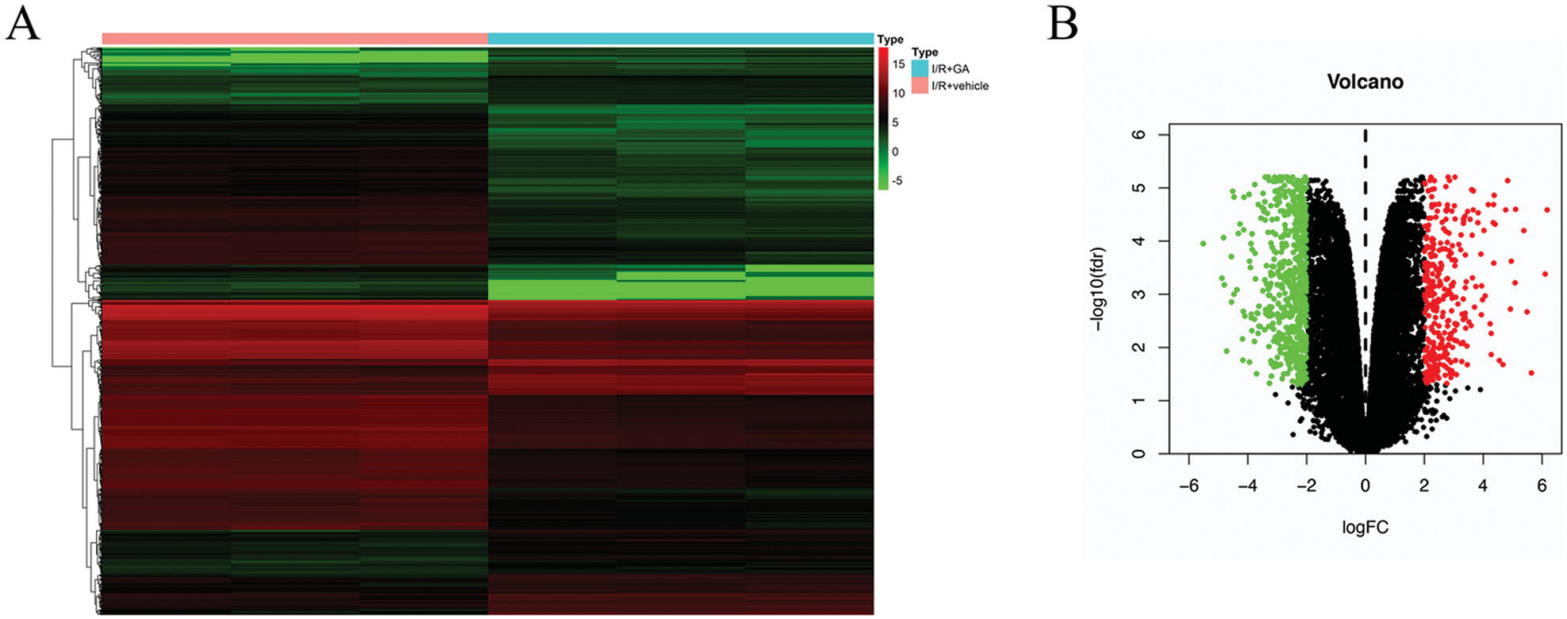
Identification of DEGs in the transcriptional level between I/R + vehicle and I/R + GA 25 mg kg^−1^ mice on day 14. (A) Heatmap of DEGs. The left three columns show I/R + vehicle samples and the right three columns represent I/R + GA samples. Red and green colors represent upregulated and downregulated expression respectively. (B) Volcano plot of DEGs. Red dots represent upregulated genes and green dots represent downregulated genes of I/R + GA group compared with the I/R + vehicle group.

To explore the mechanism of GA function *in vivo*, high-throughput RNA-seq was performed to analyze the kidney tissue with I/R injury with or without 25 mg kg^−1^ GA treatment. In the study, a twofold change or greater was used as a cutoff to select DEGs. As a result, 337 upregulated genes and 633 downregulated genes were found in the I/R + GA samples. These DEGs are shown in the heatmap and volcano plots in Figure 2. Red color represents increased gene expression, while green color denotes reduced gene expression. The results in this figure suggest that the gene expression profile in the I/R + GA group differed from that in the I/R + vehicle group.

### Enrichment analysis of DEGs

**Figure 3.**
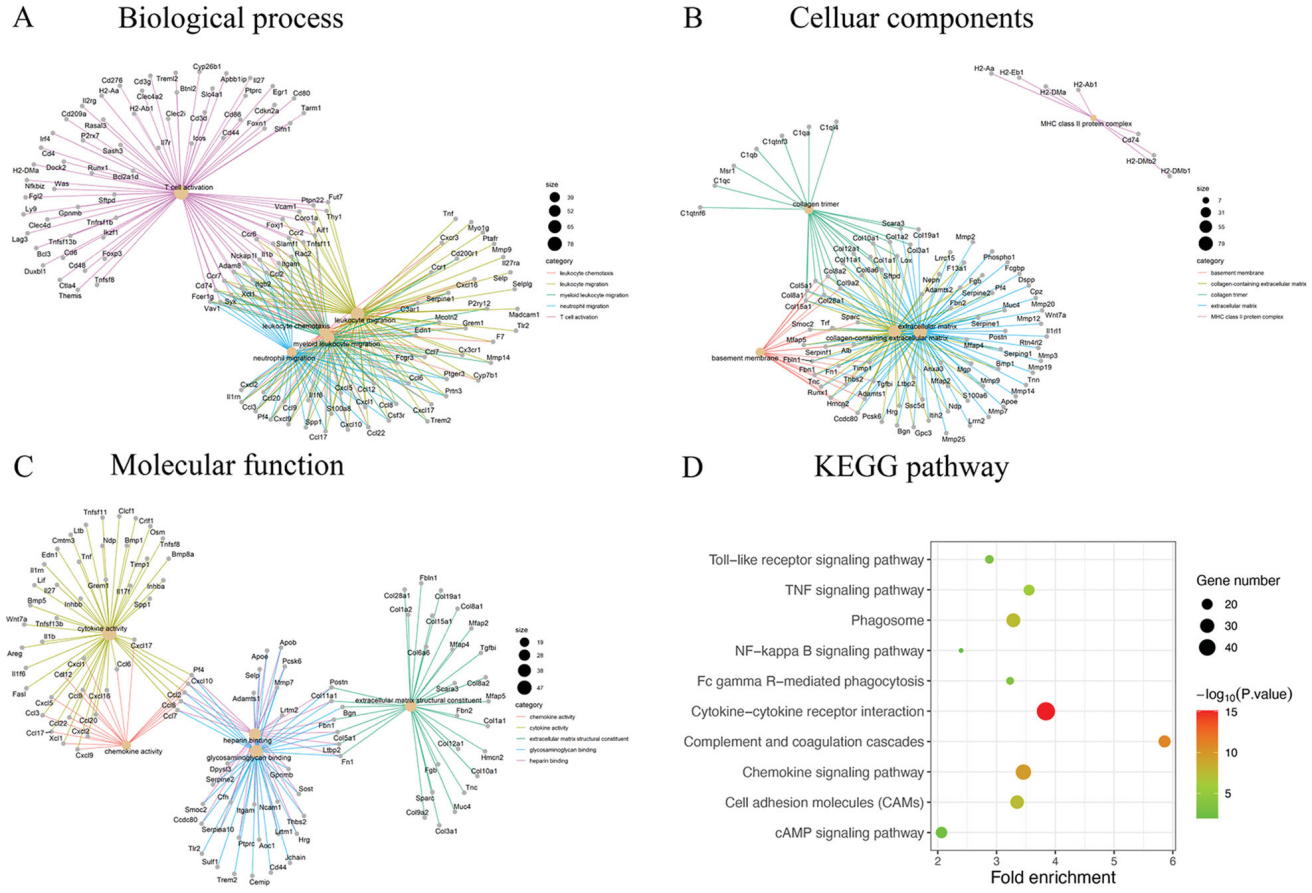
The top five GO functions for the DEGs, including (A) biological process, (B) cellular components, (C) molecular function and (D) top 10 KEGG pathways.

GO enrichment analysis was performed to detect relevant biological functions of GA treatment. The top five significantly enriched terms in BP, CC, and MF categories are demonstrated in Figure 3(A–C). In the BP ontology, we found that T cell activation was the most enriched biologic process with the highest number of DEGs (78 genes). In addition, migration-related items made up the most significant GO categories, including leukocyte migration (72 genes), myeloid leukocyte migration (52 genes), neutrophil migration (39 genes), and granulocyte migration (41 genes). In the CC ontology, most genes were related to extracellular matrix (79 genes) and collagen-containing extracellular matrix (55 genes). In the MF ontology, activity-related items accounted for two categories, containing cytokine activity (47 genes) and chemokine activity (19 genes). Similarity was observed in binding-items, containing glycosaminoglycan binding (40 genes) and heparin binding (32 genes). Extracellular matrix structural constituent (31 genes) was also another important item in MF.

To explore the underlying mechanism and potential pathways through which GA treatment mediates its protective role in renal fibrosis, the KEGG pathway analysis of 970 potential therapeutic targets was performed. The top 10 significantly enriched pathways are displayed in Figure 3(D). Among these pathways, cytokine–cytokine receptor interaction was the most prominently enriched pathway according to the gene number. Meanwhile, complement and coagulation cascades, chemokines, phagosomes, cell adhesion molecules, cAMP, TNF, Fc gamma R-mediated phagocytosis, toll-like receptor, and NF-kappa B signaling pathway were also significantly enriched, which were categorized for oxidative phosphorylation, inflammation, and proliferation.

### *Protein*–*Protein interactions network construction and hub gene selection*

**Figure 4.**
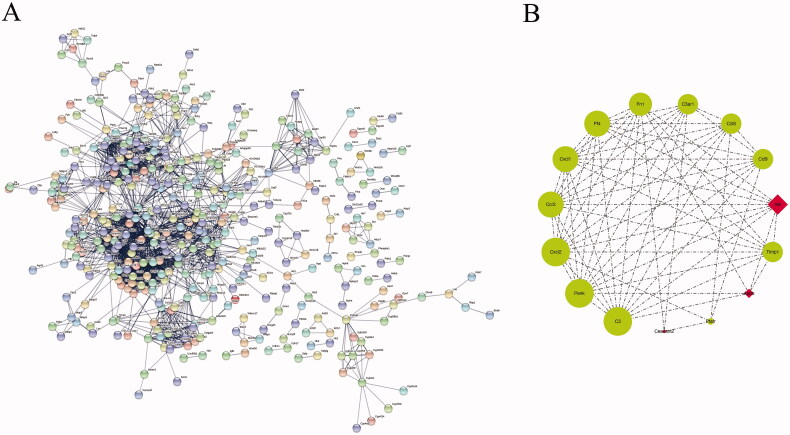
The protein–protein interaction (PPI) network, showing (A) the string interactions between the DEGs and (B) 15 hub genes. The upregulated hub genes are colored red and the downregulated hub genes are green.

The PPI network of 970 DEGs was constructed based on the online database, STRING [22,23], with the interaction score set as highest confidence (0.900). A total of 928 nodes and 2118 edges were produced (Figure 4(A)). Hub genes were calculated by the cytohubba plug-in in Cytoscape. As a result, 15 genes with highest degree ranks were selected as hub genes (Figure 4(B)).

### Verification of hub genes

**Figure 5.**
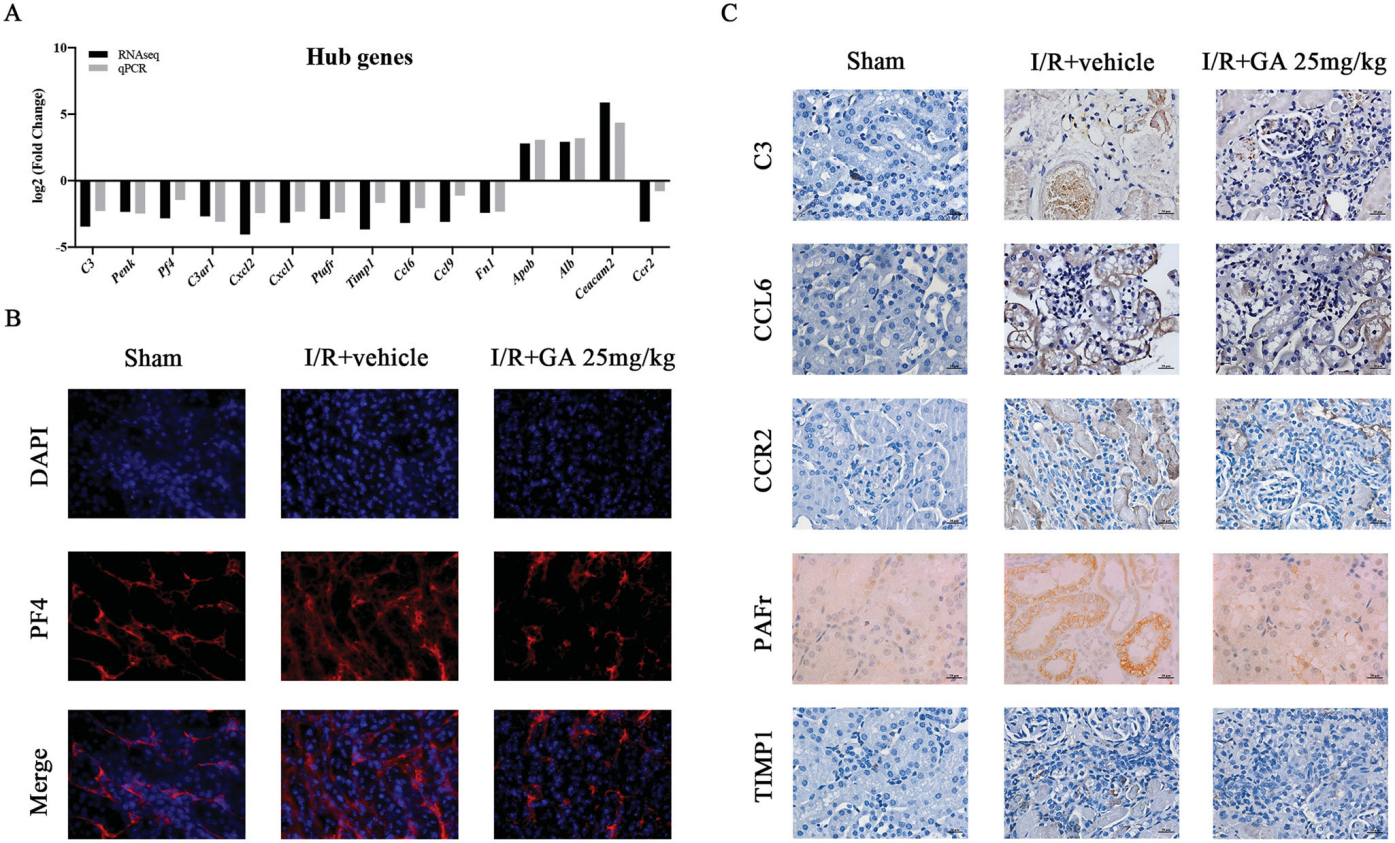
Confirmation of hub genes. (A) qPCR test of 15 hub genes compared with RNA-seq result. The trend of fold change were similar between RNA-seq and qPCR; (B) IF staining of protein PF4, which is encoded by hub gene *Pf4;* (C) IHC staining of protein C3, CCL6, CCR2, PAFr, and TIMP1, which are coded by hub genes *C3*, *Ccl6*, *Ccr2*, *Ptafr*, and *Timp1*,(×400).

The mRNA expression of hub genes was detected by qPCR to validate the results of RNA-seq. As shown in Figure 5(A), 12 genes (*C3, Penk, Pf4, C3ar1, Cxcl2, Cxcl1, Ptafr, Timp1, Ccl6, Ccl9, Fn1, Ccr2*) had significantly lower expression in kidneys of the GA treatment group, whereas the mRNA level of *Apob, Alb, and Ceacam2* were upregulated compared to the I/R + vehicle group (all *p* < .05). The outcomes of all hub genes corresponded to the changes in RNA-seq. All primers are listed in Table 1.

IHC staining of protein C3, CCL6, CCR2, PAFr, and TIMP1 exhibited the same expression trend as gene *C3*, *Ccl6*, *Ccr2*, *Ptafr*, and *Timp1* (Figure 5(C)). So did IF staining of protein PF4 (Figure 5(B)).

## Discussion

CKD and renal fibrosis are long-standing problems, which plague many scientists working in this field. The pathogenesis of progressive renal fibrosis is not fully understood; moreover, effective therapy is lacking. GA is extracted and purified from glycyrrhiza, a Chinese medicinal herb. It has been reported to have a variety of pharmacological effects. In the present study, we observed that GA exhibited antifibrotic function *in vivo*. Then, RNA-seq was applied to explore the underlying mechanism of action of GA in this renal fibrosis model. A total of 337 upregulated genes and 633 downregulated genes were identified in the I/R + GA 25 mg kg^−1^ group compared with the I/R + vehicle group. Next, we performed GO and KEGG enrichment to analyze these DEGs and the underlying signaling pathways. Then, PPI network was used to construct their interrelation and we picked out 15 hub genes, which may be the potential targets of GA in the injured kidney. Finally, hub genes were verified both in transcriptional level and translational level.

We noticed that the systemic renal function was inconsistent with single renal function. Creatinine of the insulted left kidney in I/R + vehicle group increased to about three times the baseline, while systemic SCr increased less than twice the baseline. Moreover, GA treatment effect could be detected in single kidney but not systemic. We concluded that, firstly, I/R-induced fibrosis model established successfully according to the worst left renal function and the increasing fibrosis marker. Deng et al. [24] reported similar method for modeling fibrosis. They clamped the left renal pedicle for 30 min to establish acute kidney injury model and from the day 7 after I/R injury, fibrosis could be detected on Masson and Sirius red staining, and α-SMA and Collagen-I were increased significantly in qPCR and western blot. Secondly, using single kidney creatinine rather than systemic SCr was more accurate to monitor renal function for this model. We might say the normal or mild higher creatinine of the contralateral right kidney was temporary. The function of it gradually deteriorated due to the infiltration of a large amount of inflammatory cells [25]. In the long term, both kidneys will fibrotic and SCr will be much higher without any intervention. Just like in many clinical settings, patients with kidney diseases such as glomerulonephritis will have worse and worse renal function, in the end needing renal replacement therapy.

In this study, a number of downregulated DEGs were enriched in the T cell activation process, thereby suggesting that GA treatment may be related to decreased adaptive immune response in I/R-induced renal fibrosis. T lymphocyte, an important immune cell, participates in various organ injuries. In fact, activated T cells accumulated in the kidney within just a few hours when ischemic acute renal failure occurred [26]. Classically, complete activation of T cell requires at least two signals. Signal 1 is the major histocompatibility complex (MHC) in antigen presenting cells (APC) that process antigens and present it to T cell *via* combination with T cell receptor (TCR)/CD3 complex. At the same time, B7 and CD28, on the surface of APC and T cell, respectively, recognize each other as the costimulatory signal [27]. Genes, correlated with T cell activation, were downregulated in BP analysis, such as *Icos, Cd3g, Cd3d, Cd80, Cd8, Cd3g*; *Cd3d* encodes γ and δ peptide chains, which make up protein CD3 on T cell [28]. Decreases of the two peptide chains directly influence the assembly of the TCR–CD3 complex, resulting in reduced T cell activation. In addition*, Cd80* and *Cd86* encode CD80 (B7-1) and CD86 (B7-2) of APC, respectively, which are the crucial components of the costimulatory signal in mice. Their downregulation also blocks T cell activation. ICOS is inducible and is expressed in activated T cell, but not on naïve T cell [29]. Low expression of the gene *Icos* proved that T cell activation was reduced after GA treatment. Lower activation of T cell protects against renal fibrosis. T cells are mainly divided into CD4+ and CD8+ T cells. Of note, CD4+ (but not CD8+) T cell function seems more explicit and more important in promoting renal fibrosis. Liu et al. [30] found that depleting CD4+ T cell with anti-CD4 antibody inhibited renal fibrosis in a unilateral ureteral obstruction (UUO) mouse model. Furthermore, adaptive transfer of CD4+ type 2 T helper cell (Th2) induced more severe renal fibrosis than Th1 cell, identifying CD4+ Th2 cell as a potential target in renal fibrosis.

Myeloid leukocyte migration is another important differential enrichment biologic process; specifically, the mononuclear phagocyte system has a crucial role in renal inflammation and fibrosis. Monocytes, almost entirely derived from the bone marrow, in response to inflammatory factors, are recruited in the injured kidney by chemokines. Then, monocytes transform into macrophages in the tissue. A number of chemokines contribute to macrophage recruitment and accumulation, such as CCL2, CX_3_CL1, CCL5, and CXCL16. CCL2, namely C-C motif chemokine 2, is known as monocyte chemoattractant protein 1. Alsheikh et al. demonstrated that CCL2/CCR2 was important in the early recruitment of leukocytes in rat hypertension kidney damage model [31]. In a clinical setting, Puthumana et al. found higher CCL2 level was associated with greater eGFR decline [32]. Studies have also reported that blocking CCL2 or its receptor CCR2 had a protective effect on kidney [33–35]. Pharmacological blockade of CCL2 or CCR2 have successfully entered clinical trials studying treatment of type 2 diabetes and nephropathy [36,37]. CX_3_CL1 is the only member of the CX_3_C superfamily [38], whose transmembrane region undergoes proteolytic cleavage to generate a soluble form as chemokine [39]. The study by Peng et al. [40] revealed that CX_3_CR1, the receptor of CX_3_CL1, expressed in monocytes was required for macrophage accumulation in the kidney and promoted renal fibrosis in UUO model. Similarly, other chemokines also play roles in macrophage recruitment [41–43]. Coincidentally, some genes that encode chemokines are listed in our analysis of hub genes, thereby suggesting that recruitment of immune cells to the injured focus is important for I/R-induced fibrosis and GA has the ability to reduce chemokines, and in turn, decrease the accumulation of inflammatory cells.

Macrophages have high plasticity. In *in vitro* studies, macrophages can be induced into M1 or classically activated phenotype (proinflammatory) by interferon (IFN)-γ and lipopolysaccharide (LPS) or into M2 or alternatively activated phenotype (anti-inflammatory) by interleukin (IL)-4 and IL-13 [44]. Apart from recruitment *in vivo*, there are resident macrophages in the kidney [45]. All the recruited and resident macrophages polarize to M1 or M2 subtypes according to the local microenvironment. In the I/R animal model, the infiltrated macrophage is predominantly of M1 phenotype in the early inflammatory phase, while M2 phenotype becomes predominant in the repair phase [46,47]. In impaired repair, M2 macrophage is strongly associated with renal fibrosis. Sasaki et al. found that deleting Irf4, which is a mediator of macrophage polarization to M2 subtype, could reduce renal fibrosis 4 weeks after acute kidney injury through inhibition of the PI3K/AKT signaling pathway [48]. Another study indicated that Wnt/*β*-catenin signaling pathway regulates macrophage activation and transformed them to M2 phenotype with resultant kidney fibrosis [49]. Macrophage-to-myofibroblast transition (MMT) is a well-known theory. Myofibroblast is the pivotal cell in fibrosis, contributing to collagen production and deposition. In a clinical study, CD68 + αSMA + co-expression cells were identified in active fibrotic lesions of patients’ samples, and most of these cells expressed CD206, thereby suggesting that macrophages which underwent MMT were of M2 subtype [50]. Similar results have also been found in animal studies. In addition, macrophages secrete some pro-fibrotic factors, such as matrix metalloproteinase (MMP) and platelet-derived growth factor [51–53]. The downregulated hub gene *Pf4* in our analysis, encoding platelet factor 4 (PF4), is an upstream factor of MMP-mediated fibrosis. The study by Yang et al. [54] found neutralization of PF4 could target MMP-7 and suppress macrophages in 5/6 nephrectomized mice, preventing collagen destruction in the heart tissue. All the chemokines and factors downregulated by GA treatment contributed to reduced infiltration of myeloid leukocytes and alleviated fibrosis.

Of note, our study has several limitations. First, the number of samples used for RNA-seq analysis was relatively insufficient which may have decreased the accuracy of analysis. Second, the confirmation of hub genes was only done in the rodent fibrosis model rather than human specimens, which may impede the translation of the results from mouse to human. Further investigation of the safety and effectiveness of GA treatment is needed in larger cohorts of I/R induced kidney fibrosis in humans.

## Conclusions

In summary, our research is a preliminary study on the mechanism of action of GA in the treatment of I/R-induced renal fibrosis in mice. A total of 337 upregulated and 633 downregulated DEGs were screened by bioinformatics analysis. The DEGs were enriched in decreasing immune response, cytokine–cytokine receptor interaction, and chemokine signaling pathway. Hub genes like *Pf4* provide new insight into the molecular target for GA.

## Supplementary Material

Supplemental Material

Supplemental Material

## Data Availability

The RNA-seq data have been submitted to NCBI Sequence Read Archive (SRA) with the accession number: PRJNA765889.

## Abbreviations

CKD: chronic kidney disease
GA: 18β-Glycyrrhetinic acid
I/R: ischemia/reperfusion
Nrf2: nuclear factor-erythroid 2-related factor 2
HO-1: hemoxygenase 1
AKI: acute kidney injury
HDAC2: histone deacetylases 2
BMP-7: bone morphogenetic protein-7
SCr: serum creatinine
ALT: alanine aminotransferease
AST: aspartate aminotransferase
H&E: hematoxylin-eosin
IHC: immunohistochemistry
IF: immunofluorescence
qPCR: quantitative PCR
PVDF: polyvinylidene difluoride
HRP: horseradish peroxidase
DEGs: differentially expressed genes
GO: gene ontology
KEGG: Kyoto Encyclopedia of Genes and Genomes
CC: cellular component
MF: molecular function
BP: biological processes
PPI: Protein–Protein interactions
ANOVA: one-way analysis of variance
MHC: major histocompatibility complex
APC: antigen presenting cells
TCR: T cell receptor
UUO: unilateral ureteral obstruction
Th2: type 2 T helper cell
eGFR: estimated glomerular filtration rate
IFN-γ: interferon-γ
LPS: lipopolysaccharide
IL-4: interleukin-4
MMT: macrophage-to-myofibroblast transition
MMP: matrix metalloproteinase
PF4: platelet factor 4
