# Supplementary figures and images for: Transcriptional profile changes after treatment of ischemia reperfusion injury-induced kidney fibrosis with 18β-glycyrrhetinic acid
Source: Ren Fail. 2022 Jun 14;44(1):660–71. doi: 10.1080/0886022X.2022.2061998 (PMC9225714; doi:10.1080/0886022X.2022.2061998)

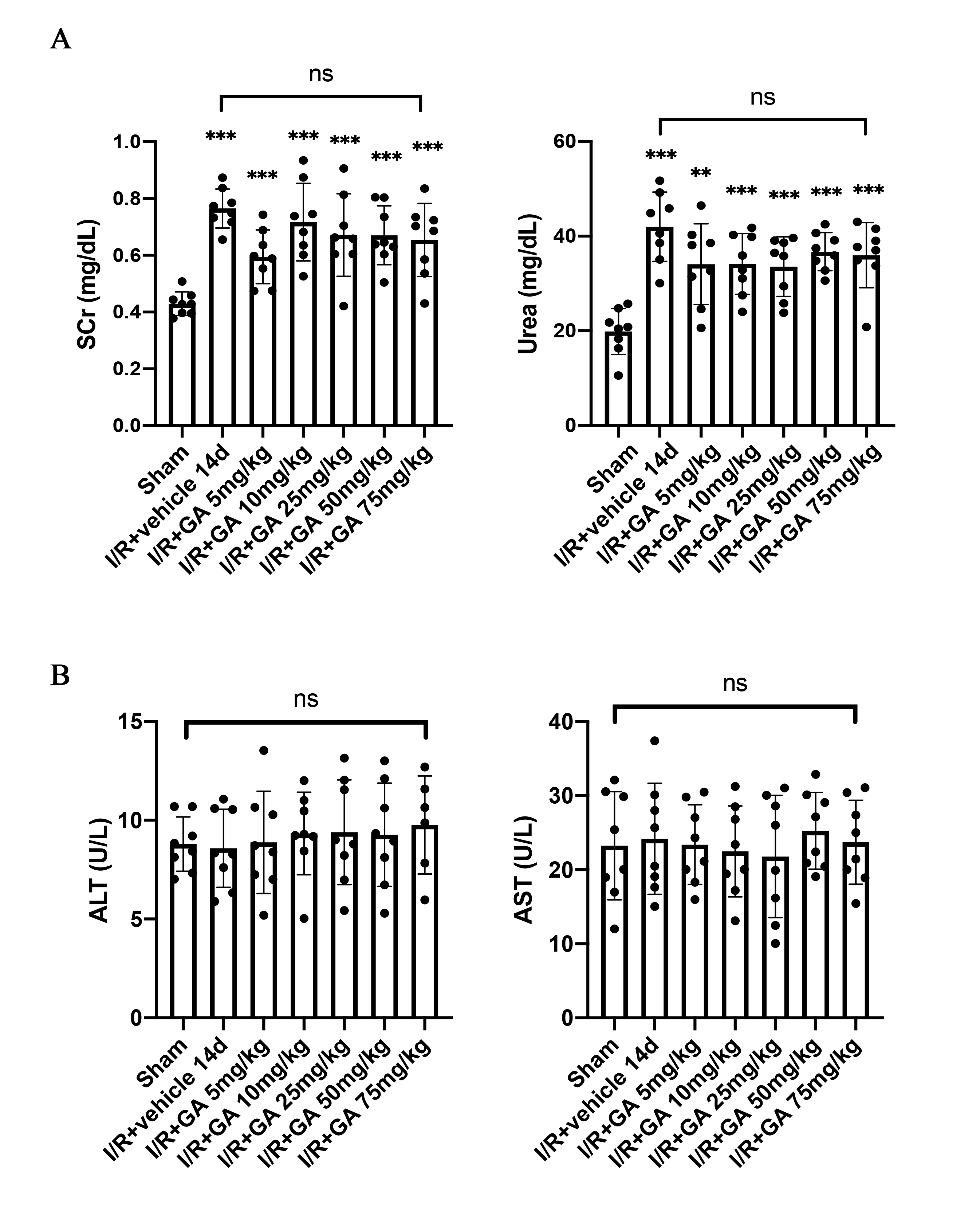

Supplement: Supplemental Material [file IRNF_A_2061998_SM4940.tif]
